# From Trash to Luxury: The Potential Role of Plant LncRNA in DNA Methylation During Abiotic Stress

**DOI:** 10.3389/fpls.2020.603246

**Published:** 2021-01-06

**Authors:** Maria Clara de Oliveira Urquiaga, Flávia Thiebaut, Adriana Silva Hemerly, Paulo Cavalcanti Gomes Ferreira

**Affiliations:** Laboratório de Biologia Molecular de Plantas, Instituto de Bioquímica Médica Leopoldo de Meis, Universidade Federal do Rio de Janeiro, Rio de Janeiro, Brazil

**Keywords:** epigenetic, non-coding RNAs, gene regulation, environmental stresses, plant breeding

## Abstract

Remarkable progress has been made in elucidating important roles of plant non-coding RNAs. Among these RNAs, long noncoding RNAs (lncRNAs) have gained widespread attention, especially their role in plant environmental stress responses. LncRNAs act at different levels of gene expression regulation, and one of these mechanisms is by recruitment of DNA methyltransferases or demethylases to regulate the target gene transcription. In this mini-review, we highlight the function of lncRNAs, including their potential role in RNA-directed DNA Methylation (RdDM) silencing pathway and their potential function under abiotic stresses conditions. Moreover, we also present and discuss studies of lncRNAs in crops. Finally, we propose a path outlook for future research that may be important for plant breeding.

## Introduction

In 1970, the central dogma of molecular biology was proposed, suggesting that the flow of information would follow the DNA to RNA to Protein (Crick, 1970). With the sequencing of the human genome, it was found that only about 3% of the genomic DNA encoded proteins and the rest was composed of the so-called “junk” DNA, including transposable elements (TEs) and highly repetitive DNA (Nowak, 1994). They also show that despite of not encoding proteins, the vast majority of human genome is transcribed into RNA. This also occurs in plant genomes. For instance, in *Arabidopsis thaliana*, the minority of its genome has the capacity of encoding proteins (Yamada et al., 2003). Nowadays, what initially was considered trash DNA became the luxury, as researchers are unraveling important roles out of the genomic non-coding sequences.

Non-coding RNAs (ncRNAs) include a huge variety of RNAs. The regulatory ncRNAs contain small RNAs (sRNAs) and long non-coding RNAs (lncRNAs) that do not encode proteins, but can generate small peptides (BenAmor et al., 2009). The best characterized are the sRNAs: microRNAs (miRNAs) and small interference RNAs (siRNAs). Several studies have highlighted the important role of sRNAs in transcriptional and post-transcriptional regulation of gene expression in plants. Although lncRNAs were previously considered to be “transcriptional noise,” emerging plant studies have Also, revealed the crucial involvement of lncRNAs in various biological processes including flowering (Fan et al., 2016), development (Zhang and Chen, 2013) and stresses responses (Sun et al., 2020).

LncRNAs are classified as ncRNAs longer than 200 nt (Ma et al., 2013). The first lncRNAs with regulatory function identified in plants was the enod40 (early nodulin 40) in Medicago, a “riboregulator” involved in plant growth (Crespi et al., 1994). With the advance of computational methods, 503 mRNA (messenger RNA)-like transcripts that appear to not encode proteins were identified in Medicago (Wen et al., 2007). Then, an increasing number of lncRNAs have been found by computational approach in different plant species (Vieira et al., 2017; Danilevicz et al., 2018). CANTATAdb^1^ is one database created to deposit these sequences, actually it collects 239,631 lncRNAs predicted in 39 species (Szcześniak et al., 2015). Although, the sequences of most lncRNAs are much less conserved than those of mRNAs, analysis of primary sequence conservation using 10 plant species revealed that the majority of lncRNAs had high sequence conservation at the intra-species and sub-species levels, in contrast to the highly diverged inter-species level (Deng et al., 2018). Moreover, lncRNAs are less expressed than mRNAs, which requires high sensitivity techniques such as RNA fluorescence *in situ* hybridization (RNA FISH), and real-time quantitative polymerase chain reaction (qRT-PCR) for the analysis of expression (Wu et al., 2020). Another feature of lncRNAs is their genomic localization, that can be located in intergenic, intronic, or coding regions, both at the sense and antisense directions (Wu et al., 2020). Interestingly, lncRNAs are regulated in response to various stimulus. Analysis of 76 lncRNAs in Arabidopsis revealed that 22 lncRNAs showed altered expression under abiotic stress (BenAmor et al., 2009). For instance, npc60 showed to be 100 times more expressed under salt stress. In cotton, lncRNA973 was increased by salt treatments and analysis by *in situ* hybridization showed that it was localized mainly to the nucleus (Zhang et al., 2019). Some studies use the subcellular localization of lncRNAs to infer their functions, since it can act both in the nucleus and cytoplasm (Karlik et al., 2019).

Although plant lncRNAs have a potential role in regulating plant responses to environmental conditions, their mechanism of function in gene regulation is poorly understood. Here, we highlight some studies that have been analyzing the importance of lncRNAs in plants. First, we included the potential roles of lncRNAs on RNA-directed DNA methylation (RdDM) silencing pathway, since many genes are methylated in response to abiotic stress. Despite showing studies on model plants, we also discuss studies of lncRNAs carried on crops, with the potential used as tools for biotechnological improvement of plants.

## LncRNAs as Precursors in RdDM Silencing Pathway

**FIGURE 1:**
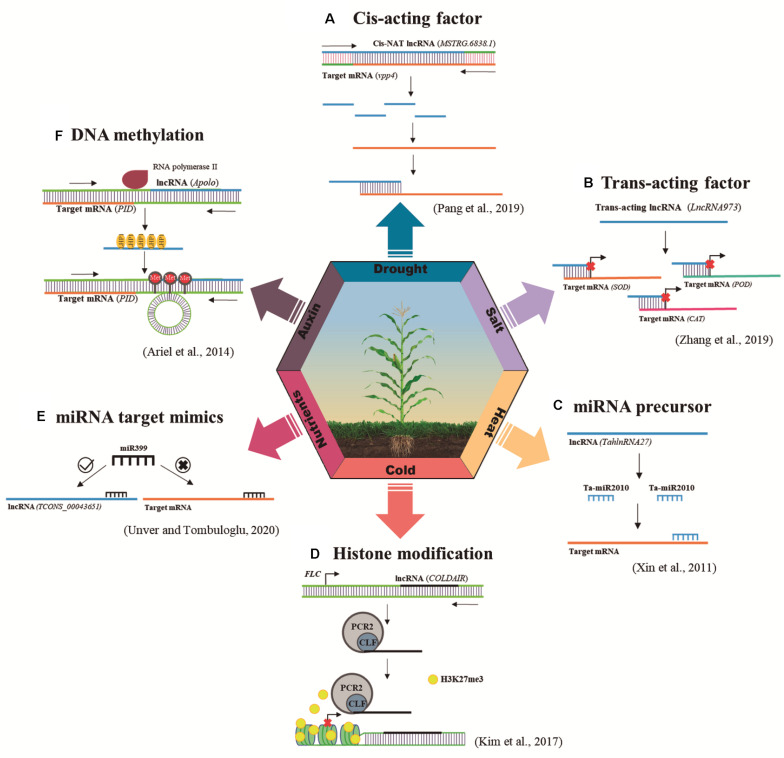
Regulatory mechanisms of plant lncRNAs in response to abiotic stresses. The main mechanisms of action triggered by lncRNAs responsive to abiotic stresses are miRNA precursor, histone modification, target mimicry, RdDM, *cis*-acting factor and *trans*-acting factor. This figure illustrates one example of each of these mechanisms. **(A)** *Cis*-acting factor: *vpp4* encoding a vacuolar (H^+^)-pumping ATPase subunit was identified as a putative target of an adjacent lncRNA *MSTRG.6838.1*. The expressions of *vpp4* and *MSTRG.6838.1* were significantly correlated in many tissues and development stages, being both repressed under drought stress, which indicates that *MSTRG.6838.1* and *vpp4* could be a promising *cis*-acting pair (Pang et al., 2019). **(B)** *Trans*-acting factor: *LncRNA973* corresponds to a *trans*-acting lncRNA responsive to salt stress, which regulates plant stress responses by modulating the expression of a series of key salt-related genes, as superoxide dismutase (SOD), peroxidase (POD), and catalase (CAT) (Zhang et al., 2019). **(C)** miRNA precursor: *TahlnRNA27*, a heat-induced lncRNA, can act as a miRNA precursor since it presents Ta-miR2010 family sequences. After 1 h of heat-treatment, *TahlnRNA27* expression was induced as well as Ta-miR2010 expression. The secondary structure and the corresponding expression pattern indicate that *TahlnRNA27* might be the precursor of Ta-miR2010 (Xin et al., 2011). **(D)** Histone modification: The repression of *FLC* by vernalization is accompanied by a series of changes in histone modifications at *FLC* chromatin, including the deposition of repressive histone markers, such as Histone H3 Lys 27 (H3K27me3). *COLDAIR* is up-regulated in response to cold, physically interacting with a component of PRC2, CURLY LEAF (CLF), for the increased enrichment of PRC2 at *FLC* chromatin to promote H3K27me3 accumulation at *FLC* (Kim et al., 2017). **(E)** miRNA target mimics: *TCONS_00043651* function as a potential natural miRNA sponge of miR399 sequence in response to boron-stress. Results obtained from barley roots analysis showed that *TCONS_00043651* was up-regulated (three times than that of control) upon boron-exposure, meanwhile miR399 expression was repressed (three times down-regulated) in the same stress conditions (Unver and Tombuloglu, 2020). **(F)** DNA methylation: *APOLO* can trigger RdDM in response to an auxin stimulus. In response to auxin, Pol II *APOLO* transcripts gradually recruit LHP1 to mediate loop formation, whereas Pol IV/V transcription triggers DNA methylation. Then, Pol II *APOLO*-LHP1 mediated loop is conformed and maintained by Pol IV/V-dependent DNA methylation to repress PID expression (Ariel et al., 2014).

LncRNAs can act as key genetic and epigenetic regulators of gene expression (Karlik et al., 2019). They may function as *cis*-acting elements by working near the site of RNA synthesis, acting directly on consecutive genes on the same strand (Zhao et al., 2020; Figure 1A); or as *trans*-acting factors by operating far from the site of synthesis (Suksamran et al., 2020; Figure 1B). LncRNAs may interfere with the binding of transcription factors to promoter regions (Csorba et al., 2014). Moreover, they can also function as miRNAs and *trans*-acting small interfering RNA (tasiRNA) precursors (Zhang et al., 2014; Fukuda et al., 2019; Figure 1C), miRNA target mimics (Shuai et al., 2014; Figure 1E) and can be processed in siRNA (Wunderlich et al., 2014). Curiously, similar to what occurs in mRNA biogenesis, the RNA polymerase II (Pol II) transcribes the majority of lncRNAs. Other RNA polymerases, such as Pol IV and Pol V that are exclusive to plants, can also act in the lncRNA generation, participating mainly in the epigenetic regulation mediated by RdDM (Wierzbick et al., 2008; Li et al., 2014). Furthermore, epigenetic mechanisms including DNA methylation (Ariel et al., 2014; Figure 1F) and histone modification (Heo and Sung, 2011; Figure 1D) are usually reported to be regulated by lncRNAs.

Plant lncRNAs play a key role in the RdDM silencing pathway. This regulatory route is based on the performance of Pol IV-dependent RNAs (P4RNAs) transcribed by Pol IV (Blevins et al., 2015; Zhai et al., 2015; Yang et al., 2016). These precursor RNAs are processed by RNA-dependent RNA Polymerase 2 (RDR2) to form double-stranded RNAs (dsRNAs), which are primarily cleaved by Dicer-like 3 (DCL3) to produce 24-nt siRNAs (Xie et al., 2004). These siRNAs are associated with Argonaute 4 (AGO4), forming AGO-siRNA complex (Holoch and Moazed, 2015). Simultaneously, lncRNAs transcribed by Pol V work as scaffold RNAs being recognized by the siRNA-AGO complex through sequence complementarity (Böhmdorfer et al., 2016). Once AGO4–siRNA–lncRNA complex is formed, it is driven to the chromatin target site together with a DNA methylation enzyme, the DNA methyltransferase domains rearranged methyltransferase 2 (DRM2) (Gao et al., 2010). This methyltransferase mediates *de novo* methylation of cytosines in all classes of sequence contexts at the target region to initiate gene silencing (Wierzbick et al., 2008). Therefore, RdDM correspond to a plant-specific *de novo* DNA methylation mechanism that requires lncRNAs as scaffold to define target genomic loci (Wierzbicki et al., 2009).

The understanding of lncRNAs role as precursors in epigenetic silencing *via* RdDM have received remarkable contributions (Chen et al., 2018, 2019). Several reports have suggested that plant lncRNAs are involved with DNA methylation performing different developmental functions such as in the regulation of embryogenesis (Chen et al., 2018), root organogenesis (Chen et al., 2019), reproduction (Ding et al., 2012), and gene silencing (Yan et al., 2018). Besides that, researchers have explored the potential of stress-regulated lncRNAs to trigger DNA methylation in response to environmental conditions. The well-characterized *AUXIN REGULATED PROMOTER LOOP (APOLO)* was identified as an auxin-induced lncRNA in Arabidopsis (Ariel et al., 2014). The double transcription of *APOLO* by Pol II and V was reported as responsible for originating a chromatin loop, which encompasses the promoter of its neighboring gene *PINOID (PID)*, a key regulator of polar auxin transport, leading to downregulation of its transcripts. Alternatively, it was proposed that *APOLO* also recognizes distant non-associated loci by R-loop formation. *APOLO*-mediated LIKE HETEROCHROMATIC PROTEIN 1 (LHP1) decoy may trigger the transcription of the target loci modulating local chromatin conformation, co-regulating auxin-responsive genes (Ariel et al., 2020). A systematic methylome study (Song et al., 2016) evaluated DNA methylation changes in *Populus simonii* submitted to salinity, osmotic and temperature stress, suggesting that, in association with miRNAs and lncRNAs, this regulatory mechanism can act in response to abiotic stresses in poplar. Ultimately, analysis in soybean roots continuously treated with high salinity solutions revealed that more than 75% of the lncRNAs identified were activated or induced in transcriptome sequencing (Chen et al., 2019).

The RdDM pathway constitutes an impressive extension of the transcriptional capacity of eukaryotic organisms, being considered the main epigenetic pathway mediated by siRNA in plants (Matzke and Mosher, 2014). The canonical RdDM pathway involves the recruitment of Pol IV to transcribe single-stranded RNAs (ssRNAs) at its target loci. The RDR2 copies the ssRNAs to produce dsRNAs. DCL3 processes dsRNAs to 24-nt siRNAs. Finally, *de novo* methylation occurs, which requires Pol V–dependent scaffold RNAs, AGO4-bound 24-nt siRNAs, and DRM2 (Mosher et al., 2008). Meanwhile, non-canonical RdDM pathways provides a link between PTGS of transposon transcripts and *de novo* methylation of transposon DNA, since it was reported that tasiRNAs and transposons are initially transcribed by Pol II, copied by RDR6 and processed by DCL2 and DCL4 into 21–22-nt siRNAs (Matzke et al., 2015). Additionally, experiments conducted in Arabidopsis *dcl1/2/3/4* mutants by Yang et al. (2016) demonstrated that DNA methylation at many of the RdDM target loci did not correlate with 24-nt siRNAs and it was completely independent of DCLs. Instead, it was observed that 25–50 nt RNAs were the main class of sRNAs generated from most RdDM loci in *dcl* plants. Interesting studies have contributed to broaden our understanding about RdDM biological functions of RdDM, reporting its involvement in regulating transposon silencing (La et al., 2011), gene expression (Lang et al., 2017), plant development (Kawakatsu et al., 2017), and biotic interactions (Satgé et al., 2016). Special attention has been given to the potential roles of DNA methylation in plant responses to a wide range of abiotic stresses, such as nutritional deficit (Secco et al., 2015), temperature (Liu et al., 2018), high salinity (Lira-Medeiros et al., 2010), and drought (Wang et al., 2016). Despite great efforts, issues such as the mechanism, biological roles and evolutionary importance of RdDM still remains to be fully elucidated, as well as the fundamental role that lncRNAs may be playing in regulating this silencing mechanism.

Functional investigations suggested the contributions of lncRNAs as essential modulators in plant responses to stresses (Figure 1). A growing body of evidence points to the great potential role for plant lncRNAs in responses to abiotic stresses *via* RdDM (Ariel et al., 2014; Yong-Villalobos et al., 2015). Given the limited number of studies, it is assumed that there is a great potential for RdDM-associated lncRNAs to be studied.

## LncRNAs as Precursors to Abiotic Stress Responses

Here, we briefly summarize recent examples of lncRNAs responsive to abiotic stresses in different plant species, with an emphasis on crop species, providing details of other mechanisms of action, in addition to the aforementioned epigenetic silencing *via* RdDM (Table 1).

**TABLE 1:** Summary of studies with abiotic stress-responsive lncRNAs in plants.

LncRNA	Stress	Plant species	Regulation mechanism	Expression	References
*IPS1*	Phosphate deficiency	*A. thaliana*	Target mimicry	Induced	Franco-Zorrilla et al., 2007
*npc536*	Salt stress	*A. thaliana*	Nat. antisense siRNAs	Induced	BenAmor et al., 2009
*npc60*	Salt stress	*A. thaliana*	Nat. antisense siRNAs	Induced	BenAmor et al., 2009
*COLDAIR*	Cold stress	*A. thaliana*	Histone modification	Induced	Heo and Sung, 2011
*TahlnRNA27*	Heat stress	*T. aestivum*	miRNA precursor	Induced	Xin et al., 2011
*TalnRNA5*	Heat stress	*T. aestivum*	miRNA precursor	Induced	Xin et al., 2011
*AtR8*	Hypoxic stress	*A. thaliana*	*Trans-*acting factor	Repressed	Wu et al., 2012
*Cis-NAT PHO1;2*	Phosphate deficiency	*O. sativa*	Translation enhancer	Induced	Jabnoune et al., 2013
*Si NAT 80*	Drought stress	*S. italica*	*Cis-*acting factor	Induced	Qi et al., 2013
*APOLO*	Auxin	*A. thaliana*	DNA demethylation	Induced	Ariel et al., 2014
*asHSFB2a*	Heat stress	*A. thaliana*	Nat. antisense siRNAs	Induced	Wunderlich et al., 2014
COOLAIR	Cold stress	*A. thaliana*	Histone modification	Induced	Csorba et al., 2014; Marquardt et al., 2014
*Lnc-173*	High-light stress	*A. thaliana*	*Cis-*acting factor	Induced	Di et al., 2014
*Lnc-225*	High-light stress	*A. thaliana*	*Cis-*acting factor	Induced	Di et al., 2014
*LincRNA1128*	Drought stress	*P. trichocarpa*	Target mimicry	Repressed	Shuai et al., 2014
*LincRNA2962*	Drought stress	*P. trichocarpa*	Target mimicry	Induced	Shuai et al., 2014
*LincRNA1039*	Drought stress	*P. trichocarpa*	Target mimicry	Induced	Shuai et al., 2014
*LincRNA20*	Drought stress	*P. trichocarpa*	Target mimicry	Induced	Shuai et al., 2014
*LincRNA2752*	Drought stress	*P. trichocarpa*	Target mimicry	Induced	Shuai et al., 2014
*LincRNA2623*	Drought stress	*P. trichocarpa*	Target mimicry	Repressed	Shuai et al., 2014
*TCONS_00056395*	Drought stress	*Z. mays*	miRNA precursor	Induced	Zhang et al., 2014
*TCONS_00082174*	Drought stress	*Z. mays*	miRNA precursor	Induced	Zhang et al., 2014
*GRMZM2G088590_T04*	Drought stress	*Z. mays*	miRNA precursor	Induced	Zhang et al., 2014
*TCONS_00037470*	Drought stress	*Z. mays*	miRNA precursor	Induced	Zhang et al., 2014
*TCONS_00012768*	Drought stress	*Z. mays*	miRNA precursor	Induced	Zhang et al., 2014
*XLOC_011965*	Cadmium stress	*O. sativa*	Unknown	Induced	He et al., 2015
*XLOC_054416*	Cadmium stress	*O. sativa*	Unknown	Induced	He et al., 2015
*XLOC_001126*	Cadmium stress	*O. sativa*	Unknown	Repressed	He et al., 2015
*XLOC_048220*	Cadmium stress	*O. sativa*	Unknown	Repressed	He et al., 2015
*TCONS_00046739*	Salt stress	*M. truncatula*	Unknown	Induced	Wang et al., 2015
*TCONS_00100258*	Salt stress	*M. truncatula*	Unknown	Induced	Wang et al., 2015
*TCONS_00118328*	Salt stress	*M. truncatula*	Unknown	Induced	Wang et al., 2015
*Os02g0250700-01*	Drought stress	*O. sativa*	Nat. antisense transcript	Repressed	Chung et al., 2016
*Os02g0180800-01*	Drought stress	*O. sativa*	Nat. antisense transcript	Repressed	Chung et al., 2016
*TCONS_00052316*	Low-nitrogen stress	*P. tomentosa*	Target mimicry	Repressed	Chen et al., 2016
*TCONS_00069233*	Low-nitrogen stress	*P. tomentosa*	Target mimicry	Repressed	Chen et al., 2016
*TCONS_00052315*	Low-nitrogen stress	*P. tomentosa*	Target mimicry	Repressed	Chen et al., 2016
*TCONS_00064021*	Low-nitrogen stress	*P. tomentosa*	*Cis-*acting factor	Repressed	Chen et al., 2016
*TCONS_00049805*	Low-nitrogen stress	*P. tomentosa*	*Cis-*acting factor	Repressed	Chen et al., 2016
*TCONS_00017288*	Low-nitrogen stress	*P. tomentosa*	Unknown	Induced	Chen et al., 2016
*TCONS_0002186*	Low-nitrogen stress	*P. tomentosa*	*Cis-*acting factor	Induced	Chen et al., 2016
*TCONS_00021860*	Low-nitrogen stress	*P. tomentosa*	Unknown	Induced	Chen et al., 2016
*c70772_g2_i1*	Drought stress	*T. turgidum*	Target mimicry	Induced	Cagirici et al., 2017
*c90557_g1_i1*	Drought stress	*T. turgidum*	Target mimicry	Induced	Cagirici et al., 2017
*TCONS_00043651*	Boron stress	*H. vulgare*	Target mimicry	Induced	Karakulah and Unver, 2017
*DRIR*	Drought and salt stress	*A. thaliana*	Unknown	Induced	Qin et al., 2017
*AK370814*	Salt stress	*H. vulgare*	*Cis-*acting factor	Induced	Karlik and Gozukirmizi, 2018
*LncRNA_082364*	Ca^2+^ -channel blocking	*T. aestivum*	*Trans-*acting factor	Induced	Ma et al., 2018
*LncRNA_047461*	Ca^2+^ -channel blocking	*T. aestivum*	*Trans-*acting factor	Induced	Ma et al., 2018
*LncRNA_074658*	Ca^2+^ -channel blocking	*T. aestivum*	*Trans-*acting factor	Repressed	Ma et al., 2018
*LncRNA_000823*	Ca^2+^ -channel blocking	*T. aestivum*	*Trans-*acting factor	Repressed	Ma et al., 2018
*LncRNA_058136*	Ca^2+^ -channel blocking	*T. aestivum*	*Trans-*acting factor	Repressed	Ma et al., 2018
*LncRNA_008977*	Ca^2+^ -channel blocking	*T. aestivum*	*Trans-*acting factor	Induced	Ma et al., 2018
*LncRNA_061738*	Ca^2+^ -channel blocking	*T. aestivum*	*Trans-*acting factor	Induced	Ma et al., 2018
*LncRNA_018111*	Ca^2+^ -channel blocking	*T. aestivum*	*Trans-*acting factor	Induced	Ma et al., 2018
*MSTRG.4636*	Heat stress	*Z. mays*	Unknown	Repressed	Lv et al., 2019
*MSTRG.38321*	Heat stress	*Z. mays*	Unknown	Repressed	Lv et al., 2019
*MSTRG.11125*	Heat stress	*Z. mays*	Unknown	Induced	Lv et al., 2019
*MSTRG.15555*	Heat stress	*Z. mays*	Unknown	Induced	Lv et al., 2019
*MSTRG.31362*	Heat stress	*Z. mays*	Unknown	Induced	Lv et al., 2019
*MSTRG.63799*	Heat stress	*Z. mays*	Unknown	Repressed	Lv et al., 2019
*AT1G34844*	Cold stress	*A. thaliana*	Nat. antisense transcript	Induced	Calixto et al., 2019
*AT3G26612 l*	Cold stress	*A. thaliana*	Nat. antisense transcript	Induced	Calixto et al., 2019
*TAS3*	Low-nitrogen stress	*A. thaliana*	*Trans-*acting factor	Repressed	Fukuda et al., 2019
*LncRNA-tomato_535*	Drought stress	*S. lycopersicum*	Target mimicry	Induced	Eom et al., 2019
*LncRNA-tomato_146*	Drought stress	*S. lycopersicum*	Target mimicry	Induced	Eom et al., 2019
*LncRNA-tomato_178*	Drought stress	*S. lycopersicum*	Target mimicry	Induced	Eom et al., 2019
*LncRNA_tomato_467*	Drought stress	*S. lycopersicum*	Unknown	Induced	Eom et al., 2019
*MSTRG.6838.1*	Drought stress	*Z. mays*	*Cis-*acting factor	Repressed	Pang et al., 2019
*VIT_216s0100n00030*	Cold stress	*V. vinifera*	*Cis-*acting factor	Induced	Wang et al., 2019
*LXLOC_027751*	Cold stress	*V. vinifera*	*Cis-*acting factor	Induced	Wang et al., 2019
*LXLOC_010422*	Cold stress	*V. vinifera*	*Cis-*acting factor	Induced	Wang et al., 2019
*VIT_202s0025n00100*	Cold stress	*V. vinifera*	*Cis-*acting factor	Induced	Wang et al., 2019
*VIT_200s0225n00020*	Cold stress	*V. vinifera*	*Trans-*acting factor	Repressed	Wang et al., 2019
*MSTRG.43964.1*	Drought stress	*C. songorica*	Target mimicry	Induced	Yan et al., 2019
*MSTRG.4400.2*	Drought stress	*C. songorica*	Target mimicry	Induced	Yan et al., 2019
*LncRNA973*	Salt stress	*G. hirsutum*	*Trans-*acting factor	Induced	Zhang et al., 2019
*TCONS_00024229*	Salt stress	*S. polyrhiza*	*Cis-*acting factor	Induced	Fu et al., 2020
*TCONS_00057092*	Salt stress	*S. polyrhiza*	*Cis-*acting factor	Induced	Fu et al., 2020
*TCONS_00018576*	Salt stress	*S. polyrhiza*	*Cis-*acting factor	Induced	Fu et al., 2020
*TCONS_00023928*	Salt stress	*S. polyrhiza*	*Cis-*acting factor	Induced	Fu et al., 2020
*TCONS_00045028*	Salt stress	*S. polyrhiza*	*Cis-*acting factor	Induced	Fu et al., 2020
*TCONS_00033722*	Salt stress	*S. polyrhiza*	Target mimicry	Induced	Fu et al., 2020
*TCONS_00018793*	Salt stress	*S. polyrhiza*	Target mimicry	Induced	Fu et al., 2020
*TCONS_ 00045706*	Salt stress	*S. polyrhiza*	Target mimicry	Induced	Fu et al., 2020
*TCONS_00057092*	Salt stress	*S. polyrhiza*	Target mimicry	Induced	Fu et al., 2020
*TCONS_ 00045512*	Salt stress	*S. polyrhiza*	Target mimicry	Induced	Fu et al., 2020
*TCONS_00051908*	Heat stress	*B. juncea*	Unknown	Induced	Bhatia et al., 2020
*TCONS_00088973*	Drought stress	*B. juncea*	Unknown	Induced	Bhatia et al., 2020
*NcM9574*	Cold stress	*M. esculenta*	*Cis-*acting factor	Induced	Suksamran et al., 2020
*NcP12248*	Cold stress	*M. esculenta*	*Cis-*acting factor	Repressed	Suksamran et al., 2020
*NcM17949*	Drought stress	*M. esculenta*	*Cis-*acting factor	Induced	Suksamran et al., 2020
*NcP456*	Cold stress	*M. esculenta*	*Trans-*acting factor	Repressed	Suksamran et al., 2020
*NcP12197*	Drought stress	*M. esculenta*	*Trans-*acting factor	Induced	Suksamran et al., 2020
*NcM15664*	Drought stress	*M. esculenta*	*Trans-*acting factor	Repressed	Suksamran et al., 2020
*LncRNA13472*	Salt stress	*S. bicolor*	Target mimicry	Induced	Sun et al., 2020
*LncRNA14798*	Salt stress	*S. bicolor*	Target mimicry	Repressed	Sun et al., 2020
*LncRNA11310*	Salt stress	*S. bicolor*	Target mimicry	Repressed	Sun et al., 2020
*LncRNA2846*	Salt stress	*S. bicolor*	Target mimicry	Repressed	Sun et al., 2020
*LncRNA26929*	Salt stress	*S. bicolor*	Target mimicry	Repressed	Sun et al., 2020
*XLOC_012868*	Drought stress	*B. napus*	Unknown	Repressed	Tan et al., 2020
*XLOC_052298*	Drought stress	*B. napus*	Unknown	Induced	Tan et al., 2020
*XLOC_094954*	Drought stress	*B. napus*	Unknown	Induced	Tan et al., 2020
*TCONS_00043651*	Boron stress	*H. vulgare*	Target mimicry	Induced	Unver and Tombuloglu, 2020
*TCONS_00061958*	Boron stress	*H. vulgare*	*Cis-*acting factor	Induced	Unver and Tombuloglu, 2020
*MtCIR1*	Cold stress	*M. truncatula*	*Cis-*acting factor	Induced	Zhao et al., 2020

A genome-wide study by Fukuda et al. (2019) reported lncRNAs that are involved in the response to low availability of nutrients in Arabidopsis, allowing the identification of 60 differentially expressed lncRNAs. Among them, *TAS3* was revealed as repressed under low-nitrogen conditions with high affinity to target *nitrate transporter 2.4* (*NRT2.4*). Similarly, a genome-wide strategy was used to identify lncRNAs differentially expressed in response to nutritional stress in poplar (Chen et al., 2016) and Arabidopsis (Franco-Zorrilla et al., 2007).

Extreme temperatures can also alter plants lncRNAs expression. In Arabidopsis, *HSFB2a* is a heat shock gene required for the gametophytic development, controlled by an antisense heat-inducible lncRNA, *asHSFB2a* (Wunderlich et al., 2014). Intriguingly, the overexpression of *asHSFB2a* represses *HSFB2a* RNA accumulation and overexpression of *HSFB2a* has a similar negative effect on *asHSFB2a* expression. Despite the lack of knowledge of the molecular mechanisms involved in this “Yin–Yang” control of sense and antisense RNA expression, the study by Wunderlich et al. (2014) showed that the vegetative and gametophytic development are impacted by this regulation of gene expression at the *HSFB2a* locus. Meanwhile, 1,614 lncRNAs were found to be differentially expressed in *Brassica juncea* under heat and drought stress conditions (Bhatia et al., 2020). Cold-responsive lncRNAs have been identified in plants such as grape (Wang et al., 2019) and Arabidopsis (Calixto et al., 2019). Both *COLDAIR* and *COOLAIR* are well-characterized examples of cold-induced lncRNAs that have been detected as regulating the vernalization process through silencing of *FLOWERING LOCUS C* (*FLC*). *FLC* encodes a MADS box transcription regulator of flowering time, repressing the induction of flowering (Heo and Sung, 2011; Marquardt et al., 2014). *COLDAIR* is transcribed from the first intron of *FLC* and physically interacts with a component of Polycomb Repressive Complex 2 (PRC2) to promote H3K27me3 accumulation at the *FLC* locus (Kim et al., 2017). *COOLAIR* is an *FLC* antisense transcript, involved in *FLC* repression by both autonomous (Tian et al., 2019) and vernalization pathways (Csorba et al., 2014), inducing H3K27me3 by recruiting plant homeo-domain (PHD)-PRC2 (Swiezewski et al., 2009).

Drought and high salinity are the main environmental conditions that adversely affect plant productivity and both can perform the same effects by overlapping genetic regulatory mechanisms. For instance, *Drought Induced lncRNA* (*DRIR*) was reported in Arabidopsis as a positive regulator of plant responses to drought and salt stress (Qin et al., 2017). Previous work identified 3 up-regulated lncRNAs under NaCl treatment (BenAmor et al., 2009) and 2,815 novel salt-responsive lncRNAs were reported in *Spirodela polyrhiza* (Fu et al., 2020). Drought-responsive lncRNAs were investigated in poplars submitted to a water deficit (Shuai et al., 2014). For example, drought induced *lincRNA2752* is a target mimic of ptc-miR169, a NF-YA transcription factor regulator. Similar results were found in drought-responsive lncRNAs identified in *Cleistogenes songorica* (Yan et al., 2019) and *B. napus* (Tan et al., 2020).

### LncRNA in Crop Plants

All findings reporting lncRNAs involvement in response to environmental stresses are particularly important in the context of crop species, since abiotic stresses are a major constraint to improve agriculture yields (Halford et al., 2015). Identification of lncRNAs during crop stress responses remains largely premature, presenting few examples (Karakulah and Unver, 2017; Pang et al., 2019).

LncRNAs have been identified as involved in nutritional homeostasis in crops such as rice (Jabnoune et al., 2013; He et al., 2015) and wheat (Ma et al., 2018). Recent reports demonstrated roles of barley lncRNAs upon excessive boron-treatment (Karakulah and Unver, 2017; Unver and Tombuloglu, 2020). Both studies suggest that boron-regulation can be cooperatively controlled by the interaction of miRNA-lncRNA-coding target transcript modules. For instance, *TCONS_00043651*, a potential miRNA sponge of miR399, was positively regulated under boron-exposure (Unver and Tombuloglu, 2020). Oppositely, miR399 expression was repressed under this stress condition.

Whereas changes in temperature often causes yield loss, heat-responsive lncRNAs were identified in wheat (Xin et al., 2011) and maize (Lv et al., 2019). The lncRNA *TahlnRNA27* was induced under heat treatment and characterized as putative miRNA precursor by presenting Ta-miR2010 family sequences (Xin et al., 2011). Similarly, 182 novel cold-responsive lncRNAs are known to be differentially expressed in cassava (Suksamran et al., 2020); whereas 2,271 lncRNAs were cold-responsive in alfalfa (Zhao et al., 2020).

Salinity stress is currently an environmental factor that most constraints agricultural productivity (Song and Wang, 2015). Studies have attempted to expand knowledge about functional mechanisms of lncRNAs in response to salt stress as well as in alfalfa (Wang et al., 2015); barley (Karlik and Gozukirmizi, 2018); cotton (Zhang et al., 2019); and sorghum (Sun et al., 2020). In particular, the *lncRNA973* overexpression had increased salt tolerance, modulating the expression of cotton salt stress-related genes (Zhang et al., 2019).

To improve crop performance in regions limited by water deficit, studies have been conducted to investigate the drought-responsive lncRNAs in crop species including foxtail millet (Qi et al., 2013); maize (Zhang et al., 2014); rice (Chung et al., 2016); wheat (Cagirici et al., 2017); tomato (Eom et al., 2019); and cassava (Suksamran et al., 2020). A recent work carried out with maize identified 124 drought-responsive lncRNAs characterized as *cis*-acting factors (Pang et al., 2019). The repressed expression correlation between *vpp4*, encoding a vacuolar (H^+^)-pumping ATPase subunit, and its adjacent lncRNA *MSTRG.6838.1* provides the idea that both could be a promising *cis*-acting pair.

## Conclusion and Perspective

Due to the rapid progress in high-throughput sequencing, several findings have significantly expanded our knowledge of lncRNA biology. However, despite the relevant results reported recently, the biological role and mechanisms of action of plant lncRNAs remain poorly understood. Further studies on lncRNAs responsive to abiotic stresses in crop species will open paths for a better understanding of their function in various processes of plant development and management of stress. It is notable in Table 1 that several lncRNAs regulated in response to abiotic stress have unknown regulation mechanisms. Remarkable progress has been made in elucidating the roles of plant lncRNAs in RdDM silencing pathway. The complexity of RdDM and its involvement in the activation of stress-responsive genes are undeniable, although more efforts are needed to understand RNA-induced DNA methylation and its function in plants, especially during abiotic stresses.

MiRNAs and lncRNAs are regulatory genes that can be targets for improving crop tolerance to abiotic stresses by using the currently advanced genome editing tools, as clustered regularly interspaced short palindromic repeats associated nucleases (CRISPR/Cas) (Zhang et al., 2020). A few successful reports on CRISPR/Cas9-based gene editing for miRNAs were published recently (Li et al., 2016; Zhou et al., 2017). The short sequences of miRNAs make it difficult to find a PAM sequence that is required for CRISPR/Cas genome editing. As more diversity of Cas proteins are identified and current Cas proteins are being continuously modified, the PAM requirement will be relaxed, and more genetic loci will become accessible by CRISPR/Cas system (Zhang and Zhang, 2020), including lncRNAs once they are already longer than miRNAs.

As the regulation for the use of genetically modified organisms (GMOs) and CRISPR-gene editing is still very tight in several countries, alternative approaches for crop breeding should be considered, such as the exogenous application of RNA molecules (Dalakouras et al., 2020). Based on successful examples of delivery of RNAs with the potential to trigger RNAi in plants (Cagliari et al., 2019; Werner et al., 2020), possible shortcomings of these methods might include optimization in application of several other types of RNA molecules, including lncRNAs, as well as grouped components of CRISPR/Cas to promote GMO independent editing events in lncRNA sequences.

## Author Contributions

MU and FT wrote the manuscript. AH reviewed the manuscript. PF contributed to design of this mini-review. All authors contributed to the article and approved the submitted version.

## Conflict of Interest

The authors declare that the research was conducted in the absence of any commercial or financial relationships that could be construed as a potential conflict of interest.

## Footnotes

1 http://cantata.amu.edu.pl, http://yeti.amu.edu.pl/CANTATA/
